# Precarity and Social Exclusion Among Older Women Living Alone

**DOI:** 10.1093/geroni/igaf122.3016

**Published:** 2025-12-31

**Authors:** Sena Odabas

**Affiliations:** Newcastle University, Newcastle upon Tyne, England, United Kingdom

## Abstract

The growing number of older women living alone presents unique challenges and opportunities. Framed by social inclusion, exclusion, and precarity, this paper examines the lived experiences of diverse women, aged 60–92, in Newcastle-upon-Tyne, UK, focusing on how life-course transitions shape socio-economic conditions in later life. Based on 26 in-depth interviews, the paper explores significant life events alongside key features of women’s social inclusion/exclusion such as social relationships, financial circumstances, and civic participation. Older women’s past experiences, present circumstances, and future expectations are analyzed through the lens of precarity.Interview data were inductively coded and interpreted using social inclusion/exclusion theory, precarity, critical gerontology, and the life-course perspective. Participants highlighted the role of evolving social relationships and life transitions in shaping their current situations and anticipated futures. Many participants associated living alone with independence and autonomy while recognizing the value of seeking advice. For most, living alone did not equate to loneliness, as their experiences were shaped by frequent social interactions and stable routines. Staying engaged and maintaining an active lifestyle were essential for well-being. While some felt financially secure, concerns about future care costs persisted. Changes in neighborhood dynamics were generally viewed positively, with strong emphasis on relationships with neighbors.This paper contributes to understanding social inclusion, exclusion, and the conditions necessary for women to live without uncertanity in later life. Further research should explore intersectional factors shaping aging trajectories to inform inclusive social policies.

